# Urinary and Generative Organs

**Published:** 1885-11-20

**Authors:** John W. S. Gouley

**Affiliations:** Surgeon to Bell Hospital, New York


### URINARY AND GENERATIVE ORGANS.

### SOME POINTS IN THE SURGERY OF THE HYPERTROPHIED PROSTATE.

By JOHN W. S. GOULEY, M.D.,
**Surgeon to Bell Hospital, New York.**

From Gaillard's Medical Journal: The idea that certain medicinal agents, taken by mouth or otherwise, can reduce the enlarged prostate though long since exploded, is from time to time revived on account of the supposed discovery of some infallible remedy. To "cure" prostatic hypertrophy, all the most likely drugs in and out of the pharmacopæa have been exhausted in vain.

A few years ago it was the fashion to inject into the substance of the prostate solutions of ergotin, iodine, and other irritants. Abscesses and their consequences were often the only rewards of these experiments.

Electricity, applied through the uretha or rectum, has had its day, and has also been found wanting, and often positively harmful.

The principal concomitant affections, complications, and sequelæ which the surgeon is called upon to treat medicinally are dyspepsia, lithuria, costiveness, constipation, sciatica and other neuroses, urethral fever, cystitis, pyelitis, pyelonephritis, polyuria, oliguria, anuria. The details of medicinal treatment need not here be given.

The surgical treatment of prostatic hypertrophy and its consequences may, with propriety, be divided into two distinct parts. First, that which relates to the mechanical means of relief of obstructed urination and its first consequence, cystitis; second, that which relates to the removal of the obstruction by operation.

### MECHANICAL MEANS OF RELIEF.

First, let me urge that all rigid catheters be discarded, except in cases of false routes, where Hey's and Meicier's methods are clearly indicated. Even when used with the greatest caution these catheters frequently do much mischief.

Among the flexible instruments now in use may be mentioned the soft vulcanized India rubber catheters, the olivary and curved gum catheters, and the elbowed and double-elbowed catheters of Mercier.

I will make a few quotations from a paper on chronic retention of urine in elderly men, which I read before the Kings County (N. Y.) Medical Society in 1882:

"I am sometimes asked what is the proper catheter, what kind of catheter should be ushd by those who cannot urinate owing to a urethro-vesical obstruction. My answer is invariably that no one kind of catheter is THE catheter. The same catheter will not suit all cases any more than the same glove will fit all hands." . . . "Let me express my own convictions as to the catheters the use of which should in general be avoided. All metallic and very rigid non-metallic catheters or soft catheters armed with stylets, should not be used under ordinary circumstances. If such instruments had never been employed to relieve retention of urine from prostatic enlargement, cases complicated with false passages, now so common, would be of extreme rarity. I have never owned what is usually called a silver prostatic catheter, nor have I ever seen a case which I could regard as requiring such an instrument. The difficulties which arise from catheterism with metallic or rigid non-metallic instruments are encountered: (1) At or near the bulbo-membranous junction of the uretha from an inordinately deep sinus bulb, undue force causing the point of the catheter to perforate mucous membranous urethra and rectum and sometimes even enter the intestine. (2) In the sinus of the prostate. This latter impediment is formed by a urethro-vesical barrier, causing a deep pouch in the prostatic sinus. In this case the same undue force may give rise to a longitudinal rent on one side or the other of the floor of the prostatic sinus, extending to or into the barrier, or else the point of the catheter penetrates and sometimes transfixes the barrier at its center. Such serious injuries are not generally inflicted with soft non-metallic instruments, though when their eyes are improperly constructed they cause considerable irritation and even erosion of the mucous membrane. Therefore, one of the prime requisites of a good catheter is an eye as small as practicable, with perfectly smooth, rounded edges. It should never have two eyes. The size of the catheter is also worthy of consideration. Nearly all those who practice auto-catheterism have a predilection for small catheters, such as Nos. 4, 5, 6 English. This is a grave error. The catheter should seldom be under No. 9, which, though it may be pliable, retains a certain degree of firmness and can be much better managed. If it seems too large the surgeon should dilate the urethra. Indeed, it is an excellent practice every few months to put the patient's urethra through a short course of dilation to No. 13 or 14, if a No. 9 is afterwards to be used, as this canal will contract from inflammation induced by the five or six daily catheterisms when these have been continued for months or years. In this condition of stenosis the introduction of the small catheter will become more and more difficult, if not impossible, and will cause hemorrhage and other mischief. In cases where false routes have been made I have advised the use of catheters as large as No. 15 English, which the patients are now introducing with the greatest ease, while before they had constantly failed with smaller instruments. Of course, in giving such advice the surgeon must be guided by the normal size of each patient's urethra."

When catheterism is to be intrusted to inexperienced persons, to nurses, or to the patients themselves, the soft vulcanized India rubber instruments are unquestionably the safest, but when they fail it is not wise to leave other catheters in their hands until they have been taught how to use them, and are made aware of the dangers attendant upon careless or rough manipulations.

Evacuative catheterism cannot be employed too soon in the management of prostatic obstruction. In ordinary cases it is an easy process attended with immediate relief, and can be repeated daily once, twice, or oftener, according to the requirements of that case.

To completely relieve a bladder from a great accumulation of stale, putrid, purulent urine at one sitting and at the same time avert the calamities (cystorrhagia, etc.) mentioned above, I suggested ten years ago the adoption of the following plan:

Let us suppose a case in which the bladder is distended so that its fundus rises above the level of the umbilicus. A catheter is introduced and one pint of urine is slowly drawn off. Immediately half a pint of warm borax solution—two grains to the ounce, with five minims of wintergreen essence and ten minims of glycerine to the ounce—is thrown in, then another pint of urine is allowed to escape through the catheter, and a half pint more of the borax solution is injected. There is already one pint less of fluid in this bladder, and what remains is mixed with a disinfectant. After this, for every half pint of diluted urine drawn off, half a pint of the borax solution is injected until the fluid that escapes from the catheter is perfectly clear. Once every four hours afterward half or a little more of the clearer contents is removed, until the bladder is entirely empty, which may take several days or a week.

Since cystitis is almost certain to be developed as a consequence of stagnation of urine, and since it is known by experience that the sudden evacuation of the distented bladder greatly aggravates this existing inflammation, which is completely relieved by gradual evacuation, but still lingers to cause rapid decomposition of the urine, and later, perhaps, phosphatic stone, it is absolutely necessary to combat it vigorossly and continuously in order to obtain over it some control. An absolute cure of the chronic cystitis accompanying prostatic hypertrophy has not yet been, and will not be, accomplished until it shall be possible to restore the urethro-vesical orifice to its normal state.

Vesical injections are used for the purpose of simply cleansing the bladder, etc. For cleansing, warm water, with the addition of a few drops of essence of wintergreen to deodorize—but any other essence will answer the purpose—is often all that is required; the density of the water to be injected is sometimes to be increased by dissolving in it a small proportion of chloride of sodium, or chlorate permanganate of potash. Phenic acid has been largely used in such cases, in solutions of various strength for the purpose of cleansing the bladder, but I fail to see in it any advantage over some of the other schools. When the urine is highly alkaline, the cleansing fluid may be mildly acidulated with nitric, hydrochloric, or phosporic acid. When the urine is purulent and slimy the bi-borate of soda or boracic acid, two grains to the ounce of warm water, seldom fails to be beneficial. After a few weeks' use of the former I have sometimes substituted the latter.

Such irrigations may be used once, or at the most, twice daily, and not more than a pint at each sitting, throwing in two or three ounces at a time. Much good may be expected by the prudent use of irrigations. They are, not unfrequently, used to excess, and this abuse of such an excellent remedy only results in disaster, especially when the fluid employed is of low density. In that case the mucous membrane becomes water-logged, its epithelium is destroyed and the cystitis utterly unmanageable.

As a modifier of the vesical mucous membrane nitrate of silver occupies the first rank; only, however, when the urine is of acid reaction. When the urine is persistently alkaline, this salt, even in very weak solution, is not tolerated, and should not be used. In certain cases of inordinate sensitiveness of the prostatic urethro-vesical orifice, the injection of an ounce or two of nitrate of silver solution, one grain to the ounce, is of great service, and its repetition once every third or fourth day for a couple of weeks renders catheterism much easier to the surgeon, and almost painless to the patient. In some other cases I have begun with very weak solutions (one-tenth of a grain to the ounce), and gradually increased the strength to two grains, seldom more, to the ounce. In these cases I have thrown into the bladder four ounces of the solution, making one such injection at each sitting.

I take this occasion to protest once more against the practice of filling the bladder with nitrate of silver solutions of twenty to thirty grains to the ounce, because this practice is still resorted to, though, I am glad to say, very exceptionably. From careful analysis of a number of cases in which this salt had been used in strong solutions, I am convinced that it should never, under any circumstances, be so employed in the treatment of cystitis.

To render catheterism less painful in cases of great irritability of the urethra and bladder, solutions of sundry substances are thereinto injected, or introduced into the rectum, such as morphia, atrophia, hyoscyamus, and more recently cocaine hydrochlorate. I have employed it in one case with some advantage.—*Quarterly Epitome.*

